# Repetition enhancement to voice identities in the dog brain

**DOI:** 10.1038/s41598-020-60395-7

**Published:** 2020-03-04

**Authors:** Marianna Boros, Anna Gábor, Dóra Szabó, Anett Bozsik, Márta Gácsi, Ferenc Szalay, Tamás Faragó, Attila Andics

**Affiliations:** 1 MTA-ELTE ‘Lendület’ Neuroethology of Communication Research Group, Eötvös Loránd University, Pázmány Péter sétány 1/C, H-1117 Budapest, Hungary; 2 Department of Ethology, Eötvös Loránd University, Pázmány Péter sétány 1/C, H-1117 Budapest, Hungary; 3 MTA-ELTE Comparative Ethology Research Group, Eötvös Loránd University, Pázmány Péter sétány 1/C, H-1117 Budapest, Hungary; 4 Department of Anatomy and Histology, University of Veterinary Medicine, István utca 2, H-1078 Budapest, Hungary; 5 University of Veterinary Medicine, István utca 2, H-1078 Budapest, Hungary

**Keywords:** Auditory system, Language

## Abstract

In the human speech signal, cues of speech sounds and voice identities are conflated, but they are processed separately in the human brain. The processing of speech sounds and voice identities is typically performed by non-primary auditory regions in humans and non-human primates. Additionally, these processes exhibit functional asymmetry in humans, indicating the involvement of distinct mechanisms. Behavioural studies indicate analogue side biases in dogs, but neural evidence for this functional dissociation is missing. In two experiments, using an fMRI adaptation paradigm, we presented awake dogs with natural human speech that either varied in segmental (change in speech sound) or suprasegmental (change in voice identity) content. In auditory regions, we found a repetition enhancement effect for voice identity processing in a secondary auditory region – the caudal ectosylvian gyrus. The same region did not show repetition effects for speech sounds, nor did the primary auditory cortex exhibit sensitivity to changes either in the segmental or in the suprasegmental content. Furthermore, we did not find evidence for functional asymmetry neither in the processing of speech sounds or voice identities. Our results in dogs corroborate former human and non-human primate evidence on the role of secondary auditory regions in the processing of suprasegmental cues, suggesting similar neural sensitivity to the identity of the vocalizer across the mammalian order.

## Introduction

The identity of the vocalizer is highly relevant in a variety of social contexts, and ethological evidence suggests that individuals excel at voice identity processing in a wide range of species (for a review see^1,2^, dogs specifically^3,4^). Recognition of the identity of the vocalizer can occur independently of speech content in humans^5^ or context in other species^6,7^. The ability to identify speech sounds, independently of who says them, is a basic building block of spoken language processing^8–10^. Neuroimaging evidence suggests that the processing of speech follows a hierarchical order in the human auditory cortex, where the different stages of increasing encoding specificity are reflected also in the neuronal topography^11–14^. In the human brain, both speech sound and voice identity processing have been localized mainly outside the primary auditory cortex, in secondary auditory regions^15–20^. The anterior part of the right superior temporal sulcus (STS) is thought to represent voice identity both in humans and non-human primates^5,19,21,22^, while the processing of speech sounds is encoded along the mid to anterior superior temporal gyrus in humans and non-human primates^11,14,15,17,23–27^.

While speech sound processing exhibits left bias^8,11,23^ or no side bias^15,28^, voice identity processing shows right hemispheric bias both in humans and in non-human primates^19,20,29^. The findings of a recent head-orienting experiment^30^ indicate similar functional asymmetry in dogs. In that study, when presented with human speech with artificially increased salience of segmental cues, dogs showed more right head-turns, indicating left hemispheric bias; while suprasegmental cues, such as intonational or speaker-related vocal information were associated with more left head-turns, indicating right hemispheric dominance. Nevertheless, orienting biases are not always coupled with hemispheric lateralization observed on the neuronal level^31^. Previous dog fMRI studies found no hemispheric bias for processing intonational cues in human speech^32^, but the neural processing of segmental or speaker-related suprasegmental cues in non-primate species has not yet been tested systematically.

The present study strives to reveal whether, similarly to primates, segmental and suprasegmental cues in human speech are processed predominantly in secondary auditory areas in dogs. We applied an fMRI adaptation paradigm that is particularly useful for studying the processing of fine changes in the speech signal^18,19,33–35^. The fMRI adaptation method is based on the observation that when brain regions sensitive to a stimulus characteristic receive repetitive input, this induces altered brain activity^36^. One apparent advantage of the adaptation paradigm for the purpose of the present study is that it can reveal neuronal specialization for subtle differences solely based on the variation in stimulus properties without requiring an active response from the subjects^36,37^.

We presented dogs with a Hungarian word “négy” [ne:ɟ] (four) that systematically varied either in its segmental content (change in the middle vowel) or in its suprasegmental content (change in voice identity). With such a manipulation we aimed at identifying the neural underpinnings of processing of segmental and suprasegmental cues found in human speech. We hypothesized that similarly to humans, the analysis of these cues in dogs takes place at higher levels of the processing hierarchy, i.e. outside the primary auditory cortex. Additionally, if these cues are processed by separate mechanisms in the dog brain, then these may also be reflected in distinct repetition effects and/or hemispheric bias.

## Methods

### Participants

Twelve dogs participated in the study (7 males, 5 females, aged from 1 to 10 years old, mean age 4.6 years, breed information: 6 golden retrievers, 5 border collies, 1 cairn terrier). The owners of dogs volunteered to take part in the project without any monetary compensation and gave written informed consent. Experimental procedures met the national and European guidelines for animal care and were approved by the local ethical committee (Pest Megyei Kormányhivatal Élelmiszerlánc-Biztonsági és Állategészségügyi Igazgatósága XIV-I-001/520-4/2012, Budapest, Hungary). The dogs were previously trained to lie motionless in the scanner for more than 8 minutes, details of the training procedure are described elsewhere^38^.

### Stimuli and procedure

Speech sound processing and voice identity processing were tested in two separate experiments, each consisting of two runs with stimulus repetition and change conditions.

**Figure 1:**
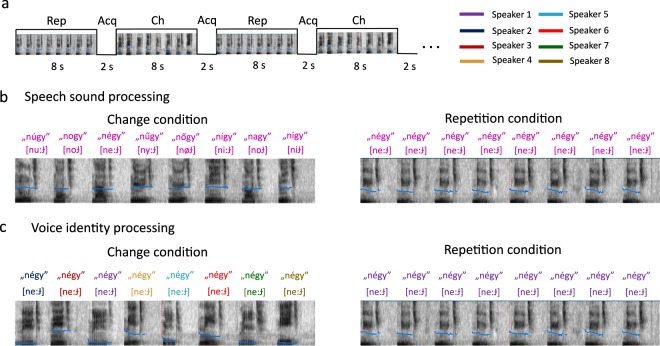
Stimulus presentation protocol. (**a**) The sparse sampling design. Rep – repetition block, Acq – MR volume acquisition, Ch – change block. (**b**) Examples of stimuli in a repetition block and in a change block in the Speech sound processing experiment spoken by one out of six (3 males and three females) speakers. (**c**) Examples of stimuli in a repetition block and in a change block in the Voice identity processing experiment.

In the speech sound processing (SSP) experiment the stimuli were 12 Hungarian CVC syllables which only differed in their middle vowel (n*V*gy [nVɟ], where V stands for: *a* [ɒ], *á* [aː], *e* [ɛ], *é* [e:], *i* [i], *í* [iː], *o* [o], *ó* [o:], *ő* [ø:], *ú* [u:], *ü* [y], *ű* [y:]). None of the 12 syllables were meaningful to any of the dogs. Ten of these syllables are nonwords and two are words in Hungarian: négy [ne:ɟ] means ‘four’ and nagy [nɒɟ] means ‘big’. We confirmed by owner interviews that neither of these words was trained to any of the dogs. The repetition condition comprised 8-second-blocks consisting of the same syllables and the change condition comprised 8-second-blocks in which the different syllables followed each other (Fig. 1b). Every change block contained 8 of the possible 12 syllables. We used 16 different recordings of every syllable per speaker. Stimuli were recorded from 3 female and 3 male speakers. Every run consisted of stimuli from a single speaker. Each dog was presented with one of the female and one of the male speakers in separate runs in a counterbalanced order.

In the voice identity processing (VIP) experiment a single syllable (*négy* [ne:ɟ]) was repeated in 8-second-blocks spoken either 8 times by the same person (repetition condition) or by 8 different persons (change condition) (Fig. 1c). We used 6 female and 6 male voices, 16 different recordings of the syllable per speaker. Every change block contained 8 voices of the possible 12. In both experiments, each recording was displayed once per run, either as part of a repetition block, or as part of a change block. The overall stimulus load was identical across runs and conditions.

In each run of both experiments, 8 additional silence blocks were added as a baseline condition, thus each run consisted of 12 repetition blocks, 12 change blocks and 8 silence blocks (32 in total) which were presented pseudo-randomly, with the criterion that two blocks from the same condition could not follow each other directly. Every dog completed the VIP experiment first and then the SSP experiment (on average 37.1 weeks later).

All stimuli were recorded from adult native speakers with neutral intonation and subsequently digitized at a 16 bit/22 kHz sampling rate and equalized for −26 dB RMS using Adobe Audition CS5.5. Stimuli were presented at a 68 dB volume level using Matlab (version 7.9) Psychophysics Toolbox 3^39^.

### Data acquisition

Scanning was carried out at the MR Research Centre of the Semmelweis University Budapest on a Philips Achieva 3 T whole body MR unit (Philips Medical Systems, Best, The Netherlands), using a Philips SENSE Flex Medium coil. For the placement of the dogs in the scanner please see^38^. Stimuli were controlled using Matlab (version 7.9) Psychophysics Toolbox 3^39^ and delivered binaurally through MRI-compatible sound-attenuating headphones (MR Confon, Magdeburg, Germany). In order to maximize the processing of the auditory stimuli a so-called sparse sampling procedure was applied (Fig. 1a) in which every stimulus block (8 s) was presented in silence and was followed by a 2 s long volume acquisition which was then immediately followed by the next block (see^32,38^ for a similar protocol). Stimulus block onset asynchrony was therefore 10 s. EPI-BOLD fMRI time series were obtained from 29 transverse slices covering the whole brain with a spatial resolution of 3.5 × 3.5 × 3.5 mm, including a 0.5 mm slice gap, using a single-shot gradient-echo planar sequence (ascending slice order; acquisition matrix 64 × 64; TR = 10000 ms, including 2000 ms acquisition and 8000 ms silent gap; TE = 36 ms; flip angle = 90°).

### Data analysis

Image pre-processing and statistical analysis were performed using SPM12 (www.fil.ion.ucl.ac.uk/spm). First, functional EPI-BOLD images were realigned and corrected for movement in the scanner. Movement parameters evidenced that each run of all dogs met the inclusion criteria of less than 3 mm movement in translation and 1 degree of rotation. Next, the mean functional image was registered manually to a custom-made individual template anatomical image^40^ and the resulting transformation matrix was applied to all realigned functional images. The normalized functional data were finally smoothed with a 4 mm (FWHM) Gaussian kernel.

Statistical parametric maps were generated using a linear combination of functions derived by convolving the standard SPM hemodynamic response function with the time series of the stimulus categories. Three different models were created. A first model (Model 1) contained data from both the speech sound processing (SSP) and the voice identity processing (VIP) experiment. Then separate models were built for each experiment, i.e. Model 2 for the SSP experiment and Model 3 for the VIP experiment. In Model 1 we included regressors for the change, repetition and silence conditions, as well as the movement parameters. We computed individual contrast images for all acoustic stimuli versus baseline (silence) for each run, which were then entered in an ANOVA for random effects group analysis. The results of this analysis served to identify peaks in the auditory cortex, which were then used to create masks for the cross-study region of interest (ROI) analyses in the experiment-specific models (see below and ROI selection).

Model 2 (SSP experiment) and 3 (VIP experiment) contained regressors for the change, repetition and silence conditions, as well as the movement parameters. Individual contrast images were computed for each acoustic (i.e. change, repetition) condition minus silence for both models (Model 2 and 3).

All results are reported at an uncorrected voxel threshold of p < 0.001, and FWE-corrected at cluster level (p < 0.05).

### ROI selection

To test for hemispheric and repetition effects in the processing of speech stimuli, a region of interest (ROI) analysis was performed. We ensured avoiding circular analysis by selecting voxels identified in either of the experiment-specific models (Model 2 or 3, see above) to extract parameter estimates form voxels of the other experiment-specific models^41,42^. We assumed that because the stimuli used in both experiments were highly similar, they would elicit overlapping activity in both tasks, and therefore the two experiments could serve as each other’s functional localizers.

First, we identified the peaks that responded most strongly to acoustic stimuli in both experiments combined (Model 1). The peaks were identified the following way. Group level activations from the all acoustic stimuli versus silence contrast of Model 1 were thresholded using the uncorrected voxel threshold of p < 0.001, and FWE-corrected at the cluster level (p < 0.05). All suprathreshold peaks, separated from each other at least by 16 mm, were selected in each hemisphere (see also^38^, Supplemental Information). Such a definition enabled us to identify activity peaks that represent anatomically distinct subregions of the auditory cortex, while ensuring that no overlap was present between the sphere masks of 8 mm in radius, that were subsequently centred on these group-level peaks.

Next, to account for the variability in the location of the individual peaks while selecting the most relevant voxels for the ROI analysis, within each sphere, the 10 most activated voxels from the all acoustic stimuli versus silence contrast of each experiment-specific model (i.e. Model 2 and 3) were identified individually in each dog^43–45^ (see Supplemenatry Tables S2 and S3).

Finally, parameter estimates (beta values) of the 10 voxels identified in the previous step were extracted from the change versus silence and repetition versus silence contrasts of the complementary model. That is, voxels identified by the all acoustic stimuli versus silence contrast of Model 2 were then used to extract parameter estimates from the change versus silence and repetition versus silence contrasts of Model 3, and vice versa. These 10 parameter estimates per ROI and condition were then first averaged within subjects, then between subjects by entering them in a mixed-model analysis of variance (ANOVA) model using the mixed procedure of IBM SPSS Statistics for Windows, version 25 (IBM Corp., Armonk, N.Y., USA). Results of the same analysis carried out with different numbers (1, 5, 15, 20, 25, 30) of selected voxels per ROI are shown in the Supplementary materials (Supplementary Table S1).

## Results

### Whole brain results

**Figure 2:**
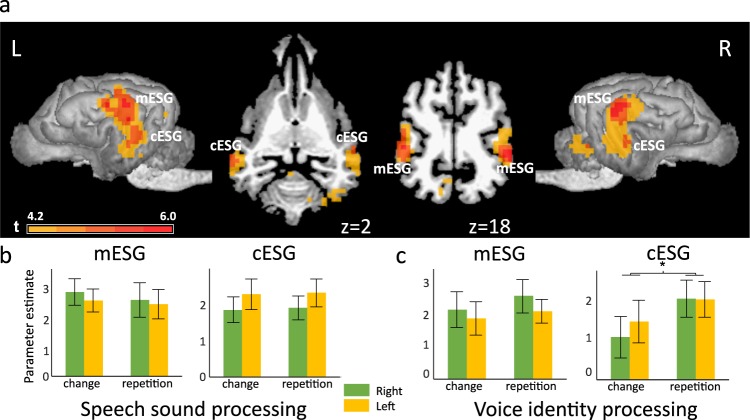
Processing of speech sounds and voice identities in dog brains. (**a**) Whole brain results of Model 1. Group level activities in the bilateral auditory cortices in response to all acoustic stimuli (speech sound processing and voice identity processing experiments combined) rendered on a template dog brain. Thresholds: p < 0.001, uncorrected for multiple comparisons at the voxel level, and an FWE-corrected threshold of p < 0.05 at the cluster level. mESG – mid ectosylvian gyrus (main peak left: −22, −16, 20; right: 24, −18, 18); cESG – caudal ectosylvian gyrus (main peak left: −26, −24, 6; right: 24, −16, 2). (**b**) The results of the ROI analysis of parameter estimates extracted from the bilateral mESG and cESG in the speech sound processing experiment. (**c**) The results of the ROI analysis of parameter estimates extracted from the bilateral mESG and cESG in the voice identity processing experiment. Error bars represent the SEM. *p < 0.05.

First, we identified regions responding to speech stimuli used in both experiments, by contrasting all acoustic stimuli with the silence condition from Model 1. On the whole-brain level, acoustic stimuli elicited extensive activity in bilateral temporal cortices encompassing the rostral, mid and caudal portions of the ectosylvian gyrus and a caudal portion of the Sylvian gyrus (Table 1 and Fig. 2). This contrast was also used to identify group level peaks around which the sphere masks were built for the ROI analysis (see below). This resulted in the selection of two peaks in the left hemisphere: one in the primary auditory cortex, the mid ectosylvian gyrus (mESG) [−22, −16, 20] and one secondary auditory region, the caudal ectosylvian gyrus (cESG) [−26, −24, 6]; and two peaks in the corresponding regions in the right hemisphere: mid ectosylvian gyrus [24, −18, 18] and caudal ectosylvian gyrus [24, −16, 2].

**Table 1:** Speech responsive auditory regions from Model 1 (all acoustic stimuli versus silence contrast).

Hemisphere	Region	Z Score	cluster size	cluster-level p_FWE-corr_	Coordinates (based on^40^)		
L	Mid ectosylvian gyrus	4.42	196	<0.001	−22	−16	20
L	Caudal ectosylvian gyrus	3.64			−26	−24	6
R	Mid ectosylvian gyrus	4.38	150	0.001	24	−18	18
R	Caudal ectosylvian gyrus	3.85			24	−16	2

Thresholds: p < 0.001, uncorrected for multiple comparisons at the voxel level, and an FWE-corrected threshold of p < 0.05 at the cluster level. The table lists all local maxima at least 16 mm apart, for each suprathreshold cluster.

The same contrast in Models 2 and 3 elicited a similar, overlapping activity in the bilateral temporal cortices (see Supplementary Fig. S1). Model 2 and 3 served to identify brain regions that participate in the analysis of segmental cues (i.e. speech sounds in the SSP experiment) and suprasegmental cues (i.e. voice identities in the VIP experiment). For this purpose, we used the change versus repetition and repetition versus change contrasts. No suprathreshold activity was found in either of these contrasts in either model.

### ROI analysis

We defined individual peaks for the ROI analysis of each experiment based on the all acoustic stimuli versus silence contrast from the other experiment (see Methods). Our underlying assumption was that using similar stimuli in the two experiments (i.e. spoken monosyllables) would result in overlapping auditory brain responses. Indeed, as evidenced by Supplementary Fig. S1, there was a considerable overlap between the auditory activity revealed in Model 2 (the SSP experiment) and Model 3 (the VIP experiment). Additional analyses confirmed that our method for the cross-localization of individual peaks is warranted. First, the average distance per dog between activity peaks in the two studies was low, ranging from 5.15 to 8.85 mm across the four ROIs (Supplementary Table S4). Second, the dispersion of the 10 most activated (and thus selected) voxels per dog in each ROI in both experiments was rather clustered, with an average distance of 3.98 mm between the individual top voxel and the remaining nine (see Supplementary Table S3).

ROI analysis was carried out to further elucidate the role of the primary and secondary auditory cortices in speech sound and voice identity processing. We expected that a repetition effect would be manifested by a selective decrease or increase in BOLD response after repeated trials. This hypothesis was tested comparing the two experiments, i.e. speech sound and voice identity processing (see Methods, Model 2 and Model 3) and parameter estimates of the different conditions extracted from the 4 ROIs have been entered in a mixed-model analysis of variance (ANOVA) model with condition (change, repetition), experiment (SSP, VIP), hemisphere (left, right) and hierarchy (primary – mESG, secondary – cESG) as within-subject fixed factors and including random effects for subjects and the interaction between the subjects and the fixed factors with an unstructured covariance structure and restricted maximum likelihood estimation.

We found a significant main effect of hierarchy (F(1,15.626) = 4.540, p = 0.049), indicating higher activity in the primary auditory cortex ROIs than in the secondary auditory cortex ROIs. Additionally, we found a significant interaction between experiment and condition (F(1,137.917) = 6.007, p = 0.015), and a significant interaction between hemisphere and hierarchy (F(1,137.917) = 5.888, p = 0.017). The first interaction showed that the activity difference between repetition and change blocks was higher in the VIP experiment, while the second showed a stronger left bias in the secondary than in the primary ROIs. Because of the interaction with the experiment factor, further analysis was carried out separately for the two experiments, i.e. for the SSP and the VIP test.

Subsequently, two mixed-model analysis of variance (ANOVA) models were created, one for each experiment. Both models included the condition (change, repetition), hemisphere (left, right) and hierarchy (primary, secondary) as within-subject fixed factors and subjects and their interactions with the fixed factors as random factors, using unstructured covariance structure and restricted maximum likelihood estimation. The SSP model showed a significant interaction between the hierarchy and the hemisphere factors (F(1,77.555) = 4.905, p = 0.040), confirming a stronger left bias in the secondary than in the primary auditory regions. The VIP model showed a marginal main effect of condition (F(1,12.343) = 4.462, p = 0.056), indicating higher activity for the repetition condition, and a significant main effect of hierarchy (F(1,11.993) = 5.325, p = 0.040), with stronger activity in the primary mESG than in the secondary cESG.

Finally, we carried out follow-up ANOVAs (two for each experiment) with condition (change, repetition) and hemisphere (left, right) as within-subject factors and subjects as random factors to investigate repetition effects in the primary (mESG) and secondary auditory (cESG) regions (Fig. 2b). There was no significant effect in either of the ROIs in the SSP experiment. In contrast, there was a significant main effect of condition (F(1,11.993) = 6.471, p = 0.026) in the secondary auditory ROI in the VIP experiment which evidenced higher activity after stimulus repetition than stimulus change.

## Discussion

In the present study we aimed at identifying the neural correlates responsible for processing human speech sounds and voice identities in dogs. Brain areas responding to human speech were identified here using various speech sounds and voices (all acoustic stimuli versus silence contrast of Model 1). These areas were highly similar to those found in previous dog fMRI studies using one speaker as stimulus^32,38,46^. Using an fMRI adaptation paradigm, we demonstrated that among speech-responsive cortical regions secondary (cESG), but not primary (mESG), auditory areas exhibited short-term repetition effects. However, repetition effects were restricted to voice identity processing, we found no repetition effects for speech sound processing.

In case of voice identity repetition, we observed a bilateral repetition enhancement effect in the cESG. Using a similar paradigm to ours, previous work has demonstrated response suppression after voice identity repetition in the human^19^ and macaque anterior superior temporal cortex^20^. Repetition suppression is often interpreted as a neuronal marker of increased processing efficiency^33,47^, while repetition enhancement may reflect novel network formation at the first encounters with unknown stimuli^48–50^. Although previous reports^19,20^, just as the present study, used unfamiliar stimuli, one key difference that could have contributed to different repetition effect, is whether the vocalizer and listener species were the same or not. Comparative neuroimaging evidence^38^ shows that dog voice-sensitive areas, just like human and macaque voice-sensitive areas^51^, prefer conspecific vocalizations. The dog auditory cortex is therefore not as tuned to human vocalizations as the human auditory cortex is. While dogs participating in our study are indeed surrounded by a human environment and thus are constantly exposed to speech, the number of individuals they listen to is typically much more limited than the number of individuals humans listen to. It is possible that while upon hearing a new voice, humans can readily position that voice in a voice similarity space^29,52^, dogs may not possess such a representational space for human voices. If this is the case, then the processing of unfamiliar human voices may entail different neural computations in dogs than in humans and thus the elevated neuronal activity might reflect additional internal operations of building a new representation. Conversely, it is possible that the repetition suppression for unfamiliar voices is not specific to human listeners, but is specific to conspecific vocalizations. To confirm this speculation, further experiments are necessary.

The observed repetition effect was manifested in the secondary, not the primary auditory cortices. As evidenced by our ROI analysis, altered brain activity after stimulus repetition was only present in the cESG, a region located anterior/ventrally to the primary auditory cortex in dogs. A multistage processing hierarchy of speech in humans is often described as a cascade comprising increasingly complex representations, where after a basic acoustic analysis higher order phonetic and word-level information is processed separately from speaker-related information^5^. Our findings indicate a functional distinction between primary and secondary auditory regions in dogs. The mESG showed a strong response to all acoustic stimuli, but remained insensitive to stimulus repetitions. The cESG, in contrast, exhibited stronger activity for processing repetition blocks compared to change blocks. Our results, combined with previous findings demonstrating stimulus specificity in secondary auditory cortices in dogs^32^, indicate that the processing of speech involves several stages of analysis in the dog auditory cortex, and that suprasegmental cues may be processed by higher level, non-primary brain regions.

Consistent with our results, human and non-human primate data suggests that the anterior part of the right superior temporal lobe is involved in representing individual voices^19,20,29^. This has led to the formation of a neurocognitive theory that the anterior temporal lobe hosts a set of regions and mechanisms that allow humans and macaques to identify individuals^22,51,53^. In this brain network, the anterior superior temporal cortex plays a similar role to the fusiform face area (FFA)^54^, a category specific region located in the ventral visual stream, that is involved in the processing of faces. The so-called temporal voice areas containing the anterior temporal cortex exhibit a preference to process conspecific vocalizations over other sounds in humans^55^, macaques^20^ and marmosets^56^. Previously we have identified a functional analogue of conspecific-preferring voice areas in dogs^38^, and here we extended these results by demonstrating that, similarly to humans and macaques, this anterior temporal region (in dogs, this is anatomically defined as the caudal portion of the ectosylvian gyrus) is involved in the processing of individual voice identity^51^.

The voice identity processing in our study did not show any hemispheric bias in the cESG, but resulted in overall repetition enhancement. A considerable body of human^19,29,57,58^ and non-human primate^20,59^ studies points to a right hemispheric bias for analysing the identity of the vocalizer. Using a similar paradigm as here, Belin and Zatorre^19^ in humans demonstrated repetition suppression in the anterior part of the right superior temporal gyrus after stimuli were repeated by the same speaker. However, bilateral repetition suppression effects in response to voice repetition were also reported in the posterior superior temporal sulcus in humans^18^. In dogs, earlier behavioural studies on the lateralization of the processing of vocalizations yielded contradicting results, reporting both left^60^ and right hemispheric^61^ dominance for conspecific vocalizations, and right bias for processing interspecific vocalizations (cats and humans^61^). Ratcliffe and Reby^30^ found left orienting asymmetry when presenting dogs with human vocalizations with artificially increased saliency of speaker-related suprasegmental information, indicating right hemispheric bias in dogs for processing human voices. Note however, that all these dog studies focused on vocalization processing in general, and did not test voice identity effects. Our results suggest that in dogs, unlike in humans, voice identity processing may not show a laterality bias.

In contrary to the voice identity processing experiment, we did not find any repetition effect in the processing of speech sounds neither in the primary nor in the secondary auditory cortex. It is not clear why a repetition effect was not present in our sample. Previous behavioural studies on speech sound processing in dogs established that they are able to discriminate the phonetic content of meaningful words^30,62^. Note however, that both studies contrasted the processing of meaningful commands and their degraded versions, while here we used meaningless syllables for the dogs. A more relevant result comes from an early electrophysiological study by Adams and colleagues^63^, who presented anaesthetised 15-week-old border collies with CV syllables along the/ga/-/ka/continuum and recorded the evoked auditory responses. They found categorical speech sound discrimination in the right primary auditory cortex. Here, we found neither evidence for discrimination nor sensitivity to speech sounds in any of the brain areas tested. This is in line with previous works from Belin and Zatorre^19^, who in a similar paradigm to ours, also failed to demonstrate repetition effects in speech sound processing in humans. It might be that the present task was not sensitive enough to induce such an effect.

Regarding laterality, we found a stronger left bias in dogs in the secondary than the primary auditory cortex for processing speech stimuli. This is in line with the study by Ratcliffe and Reby^30^ who, in a head-turning paradigm, found that dogs oriented significantly more with their right ear in response to speech with artificially enhanced saliency of segmental cues, indicating a contralateral (left) bias for processing speech sounds. Although statistically only present in form of an interaction effect, our results might demonstrate the possible neural underpinnings of this behavioural effect. In the present study the lateralization bias was more apparent in the SSP experiment, and it was absent in the VIP experiment, but we found no effect of condition, thus whether this bias is of segmental nature remains unclear.

There are a few possible limitations of our study. First, we used a fast miniblock design with short inter-stimulus intervals (to maximize the number of trials we could present using a limited run length for which the dogs were trained). As a consequence, we cannot exclude the possibility of an across-block effect between a repetition block and the same syllable or voice identity in a subsequent change block, potentially causing a repetition effect also in the change block, making it more similar to repetition blocks, and thus reducing design sensitivity. However, we believe that the on average 14 s lag between the repetition block and the onset of the same syllable or voice identity in the subsequent change block was long enough not to cause a considerable short term repetition effect on the change block. While longer term (across-block) repetition effects do exist, these effects are thought to be dissociable from short term (within-block) repetition effects, and the two are thought to be separately modifiable^64^. Second, due to the fixed order of the experiments (first VIP, then SSP), we cannot exclude that the lack of repetition effects in the SSP experiment is related to an overall habituation of the participating dogs to the stimuli. Nevertheless, while stimuli in the two experiments were indeed similar, the actual stimulus overlap across the experiments was minimal, and we believe that an average gap of 37.1 weeks between the scanning sessions for the two experiments was long enough to make a considerable order effect on the SSP experiment results improbable.

## Conclusions

We revealed a secondary auditory region in the dog brain – the caudal ectosylvian gyrus – that is involved in the processing of voice identities. This provides neural evidence for dogs’ sensitivity for suprasegmental cues in human speech. The previously identified shared neural mechanisms across humans and monkeys for voice identity processing^19,20^ gave rise to a view that the anterior temporal lobe hosts a brain network implicated in the identification of individuals^21,22,51^. Here we demonstrate that a functional analogue of such a network might also be present in an evolutionarily distant non-primate species, the dog.

## Supplementary information

Supplementary information.
