# Supplementary material for: Repetition enhancement to voice identities in the dog brain
Source: Sci Rep. 2020 Mar 4;10:3989. doi: 10.1038/s41598-020-60395-7 (PMC7055288; doi:10.1038/s41598-020-60395-7)
Supplement: Supplementary file 1 — Supplementary information. [file 41598_2020_60395_MOESM1_ESM.pdf]

Repetition enhancement to voice identities in the dog brain

Supplemental Information

Marianna Boros[1,2], Anna Gábor[1,2], Dóra Szabó[2], Anett Bozsik[1,5], Márta Gácsi[2,3], Ferenc Szalay[4], Tamás Faragó[2] & Attila Andics[1,2]

[1] MTA-ELTE 'Lendület' Neuroethology of Communication Research Group, Hungarian Academy of Sciences – Eötvös Loránd University, H-1117 Budapest, Pázmány Péter sétány 1/C, Hungary.

[2] Department of Ethology, Eötvös Loránd University, H-1117 Budapest, Pázmány Péter sétány 1/C, Hungary.

[3] MTA-ELTE Comparative Ethology Research Group, Hungarian Academy of Sciences – Eötvös Loránd University, H-1117 Budapest, Pázmány Péter sétány 1/C, Hungary.

[4] Department of Anatomy and Histology, University of Veterinary Medicine, H-1078 Budapest, István utca 2, Hungary.

[5] University of Veterinary Medicine, H-1078 Budapest, István utca 2, Hungary.

Keywords: secondary auditory cortex, voice identity processing, fMRI adaptation, dog, comparative neuroimaging

Correspondence to: attila.andics@gmail.com, marianna.cs.boros@gmail.com

Model	full model with experiment, hierarchy, hemisphere and condition as fix factors						VIP experiment				SSP experiment		VIP cESG	
effects or interactions of interest	condition x experiment		hierarchy		hemisphere x hierarchy		condition		hierarchy		hemisphere x hierarchy		condition	
	F	*p*	F	*p*	F	*p*	F	*p*	F	*p*	F	*p*	F	*p*
1 vox	3.757	*0.054*	2.94	*0.105*	2.604	*0.109*	4.47	*0.056*	5.346	*0.037*	3.955	*0.049*	6.257	*0.028*
5 vox	5.402	*0.021*	3.685	*0.071*	8.4	*0.004*	4.058	*0.072*	5.458	*0.039*	6.125	*0.017*	6.633	*0.024*
10 vox	6.077	*0.015*	4.54	*0.049*	5.888	*0.017*	4.462	*0.056*	5.325	*0.04*	4.905	*0.03*	6.471	*0.026*
15 vox	5.721	*0.018*	3.779	*0.076*	5.133	*0.025*	4.109	*0.066*	4.71	*0.051*	4.811	*0.033*	5.804	*0.033*
20 vox	6.733	*0.011*	4.107	*0.066*	5.832	*0.017*	4.179	*0.065*	4.54	*0.054*	5.524	*0.022*	5.718	*0.036*
25 vox	6.898	*0.01*	4.54	*0.054*	4.55	*0.035*	3.952	*0.069*	4.367	*0.058*	3.649	*0.062*	5.849	*0.032*
30 vox	7.184	*0.008*	4.705	*0.051*	4.784	*0.3*	3.814	*0.076*	4.207	*0.063*	3.295	*0.076*	5.695	*0.034*
8 mm smooth	11.799	*0.001*	4.709	*0.051*	1.851	*0.176*	3.512	*0.081*	5.655	*0.033*	2.235	*0.141*	4.298	*0.066*
Quasi-event	7.09	*0.009*	6.401	*0.021*	7.069	*0.009*	4.624	*0.053*	6.897	*0.022*	5.157	*0.026*	6.787	*0.023*

Supplementary Table S1. Results of additional ROI analyses. 1-30 vox – after preprocessing, parameter estimates extracted from and averaged over the top 1 to 30 voxels in each ROI (see the main text for details). 8 mm smooth – data smoothed with an 8 mm FWHM Gaussian kernel, parameter estimates extracted from and averaged over the top 10 voxels in each ROI. Quasi-event – only the middle two stimuli of an experimental block (4th and 5th) are modelled in Model 2 and 3, data preprocessed as described in the manuscript, parameter estimates extracted from and averaged over the top 10 voxels in each ROI.

Subject	Voice identity processing				Speech sound processing			
	left mESG (-21, -15, 16)	left cESG (-23, -22, 6)	right mESG (22, -18, 16)	right cESG (23, -18, 1)	left mESG (-21, -18, 18)	left cESG (-24, -22, 7)	right mESG (22, -18, 16)	right cESG (23, -18, 0)
Barney	3.62	1.86	5.29	7.81	2.96	4.23	2.51	4.57
Bodza	5.75	7.97	4.44	7.88	2.62	2.22	1.52	2.76
Bran	6.9	6.98	3.93	4.87	2.74	4.96	5.67	4.67
Grog	5.75	4.08	7.02	5.35	8.38	6.52	2.18	4.67
Guru	3.66	5.82	1.93	7.96	6.67	5.32	5.78	8.57
Kefír	3.62	5.82	3.72	6.63	2.74	4.96	6.64	9.21
Kope	3.98	2.73	6.13	4.64	2.03	8.68	6.67	4.43
Manka	5.64	5.41	6.64	7.06	4.01	8.52	4.17	5.17
Maverick	1.31	6.98	5.21	6.63	4.75	3.56	4.82	7.14
Maya	4.95	4.51	5.92	8.41	2.18	5.44	6.06	7.5
Nia	5.73	4.45	4.84	6.37	2.96	7.55	6.06	3.32
Sander	4.79	5.95	5.09	7.96	2.96	5.71	1.52	1.55
mean	*4.64*	*5.21*	*5.01*	*6.80*	*3.75*	*5.64*	*4.47*	*5.30*

Supplementary Table S2. Distances of individual peaks to group centroids. Centroids of the 12 individual peak coordinates of the All acoustic stimuli vs. Silence contrast in all 4 ROIs (left and right primary and secondary cortices) in the two experiments (Speech sound and Voice identity processing). Individual distances are calculated from each individual peak coordinate to the centroid for each ROI and experiment.

Voice identity processing

Subject	left mESG coord (x , y , z)	left mESG mean D (min , max)	left cESG coord (x , y , z)	left cESG mean D (min , max)	right mESG coord (x , y , z)	right mESG mean D (min , max)	right cESG coord (x , y , z)	right cESG mean D (min , max)	*subject mean*
Barney	(-22 , -20 , 16)	2.53 (2 , 4)	(-22 , -20 , 10)	2.64 (2 , 5)	(24 , -18 , 18)	3.91 (2 , 9)	(16, -16, 2)	4.58 (2 , 12)	*3.41*
Bodza	(-20 , -20 , 18)	3.82 (2 , 7)	(-24 , -20 , 8)	2.85 (2 , 5)	(22 , -20 , 16)	4.76 (2 , 9)	(20, -12, -2)	3.31 (2 , 7)	*3.68*
Bran	(-22 , -16 , 20)	3.08 (2 , 5)	(-24 , -18 , 10)	6.74 (2 , 12)	(20 , -16 , 12)	2.53 (2 , 4)	(26, -20, -2)	2.55 (2 , 5)	*3.72*
Grog	(-20 , -10 , 20)	2.69 (2 , 4)	(-22 , -28 , 6)	2.44 (2 , 4)	(20 , -18 , 16)	4.36 (2 , 9)	(26, -16, 6)	5.76 (2 , 12)	*3.81*
Guru	(-18 , -22 , 22)	2.53 (2 , 4)	(-26 , -18 , 4)	3.92 (2 , 7)	(20 , -24 , 16)	2.51 (2 , 4)	(24, -16, -6)	8.27 (2 , 16)	*4.3*
Kefir	(-22 , -20 , 20)	2.94 (2 , 5)	(-24 , -18 , 10)	3.23 (2 , 7)	(22 , -12 , 16)	3.46 (2 , 7)	(24, -20, 8)	3.29 (2 , 8)	*3.23*
Kope	(-22 , -20 , 18)	2.44 (2 , 4)	(-26 , -30 , 10)	4.31 (2 , 8)	(20 , -22 , 22)	2.81 (2 , 5)	(24, -22, 4)	2.76 (2 , 5)	*3.08*
Manka	(-22 , -22 , 18)	2.44 (2 , 4)	(-24 , -30 , 10)	3.74 (2 , 11)	(24 , -20 , 20)	5.31 (2 , 8)	(26, -20, 8)	10.45 (2 , 15)	*5.48*
Maverick	(-24 , -18 , 14)	2.88 (2 , 5)	(-24 , -20 , 10)	6.12 (2 , 13)	(22 , -14 , 18)	2.67 (2 , 5)	(24, -20, 8)	7.51 (2 , 14)	*4.79*
Maya	(-22 , -18 , 16)	2.46 (2 , 3)	(-22 , -22 , 12)	4.15 (2 , 11)	(24 , -22 , 12)	2.78 (2 , 5)	(22, -12, -4)	2.95 (2 , 5)	*3.08*
Nia	(-24 , -18 , 20)	3.04 (2 , 5)	(-26 , -20 , 0)	3.1 (2 , 5)	(24 , -22 , 12)	2.97 (2 , 7)	(26, -22, 6)	5.44 (2 , 10)	*3.63*
Sander	(-22 , -20 , 16)	4.66 (2 , 8)	(-26 , -20 , 2)	3.89 (2 , 8)	(22 , -20 , 16)	3.39 (2 , 6)	(24, -16, -6)	7.73 (3 , 11)	*4.91*
group mean		*2.95*		*3.92*		*3.45*		*5.37*	*3.92*

Speech sound processing

Subject	left mESG coord (x , y , z)	left mESG mean D (min , max)	left cESG coord (x , y , z)	left cESG mean D (min , max)	right mESG coord (x , y , z)	right mESG mean D (min , max)	right cESG coord (x , y , z)	right cESG mean D (min , max)	*subject mean*
Barney	(-22 , -18 , 14)	3.09 (2 , 6)	(-22 , -22 , 6)	5.62 (2 , 10)	(16 , -16 , 2)	2.46 (2 , 3)	(26 , -22 , 0)	2.53 (2 , 4)	*3.42*
Bodza	(-22 , -10 , 16)	2.71 (2 , 5)	(-22 , -30 , 4)	2.59 (2 , 4)	(20 , -12 , -2)	3.52 (2 , 7)	(24 , -20 , -2)	5.48 (2 , 11)	*3.57*
Bran	(-18 , -10 , 18)	2.51 (2 , 4)	(-24 , -20 , 0)	5.48 (2 , 11)	(26 , -20 , -2)	2.62 (2 , 4)	(22 , -20 , 4)	3.43 (2 , 6)	*3.51*
Grog	(-22 , -10 , 16)	7.1 (2 , 12)	(-22 , -26 , 8)	2.46 (2 , 3)	(26 , -16 , 6)	2.73 (2 , 5)	(22 , -20 , -4)	6.1 (2 , 14)	*4.59*
Guru	(-20 , -16 , 20)	4.36 (2 , 15)	(-24 , -18 , 10)	2.98 (2 , 5)	(24 , -16 , -6)	7.26 (3 , 11)	(26 , -20 , 8)	11.57 (3, 15)	*6.54*
Kefir	(-24 , -16 , 14)	3.81 (2 , 8)	(-24 , -18 , 10)	4.34 (2 , 8)	(24 , -20 , 8)	3.16 (2 , 7)	(22 , -10 , 4)	2.82 (2 , 4)	*3.53*
Kope	(-22 , -18 , 20)	2.71 (2 , 5)	(-26 , -24 , 6)	10.23 (2 , 15)	(24 , -22 , 4)	3.54 (2 , 5)	(24 , -20 , -4)	3.44 (2 , 11)	*4.98*
Manka	(-20 , -20 , 20)	2.37 (2 , 3)	(-24 , -28 , 6)	3.2 (2 , 5)	(26 , -20 , 8)	5.3 (2 , 9)	(26 , -16 , -4)	4.86 (2 , 13)	*3.93*
Maverick	(-22 , -16 , 18)	2.84 (2 , 5)	(-24 , -20 , 0)	3.42 (2 , 9)	(24 , -20 , 8)	2.68 (2 , 5)	(24 , -22 , 6)	2.71 (2 , 5)	*2.91*
Maya	(-24 , -20 , 16)	2.6 (2 , 4)	(-24 , -22 , 2)	4.19 (2 , 9)	(22 , -12 , -4)	4.85 (2 , 13)	(22 , -12 , -4)	3.69 (2 , 11)	*3.83*
Nia	(-22 , -18 , 22)	6.53 (3 , 9)	(-26 , -24 , 10)	6.71 (2 , 13)	(26 , -22 , 6)	3.46 (2 , 7)	(22 , -16 , -2)	2.53 (2 , 4)	*4.8*
Sander	(-22 , -16 , 12)	3.75 (2 , 7)	(-22 , -24 , 12)	2.53 (2 , 4)	(24 , -16 , -6)	3.05 (2 , 5)	(22 , -18 , 0)	2.6 (2 , 4)	*2.98*
group mean		*3.69*		*4.47*		*3.71*		*4.31*	*4.04*

Supplementary Table S3. Dispersion of voxels of interest per subject. Individual peak coordinates of the All acoustic stimuli vs. Silence contrast in all 4 ROIs (left and right primary and secondary cortices) in the two experiments (Speech sound and Voice identity processing). Mean distances (D) are calculated from the distance of the top individual voxel to each of the 9 remaining voxels, rounded min and max distances are given in bracket (mm).

	Individual peak coordinates											
	left mESG			left cESG			right mESG			right cESG		
Subject	SSP	VIP	D	SSP	VIP	D	SSP	VIP	D	SSP	VIP	D
Barney	(-22 , -20 , 16)	(-22 , -18 , 14)	2.83	(-22 , -20 , 10)	(-22 , -22 , 6)	4.47	(24 , -18 , 18)	(22, -22, 12)	7.48	(26 , -22 , 0)	(16, -16, 2)	11.83
Bodza	(-20 , -20 , 18)	(-22 , -10 , 16)	10.39	(-24 , -20 , 8)	(-22 , -30 , 4)	10.95	(22 , -20 , 16)	(22, -20, 12)	4	(24 , -20 , -2)	(20, -12, -2)	8.94
Bran	(-22 , -16 , 20)	(-18 , -10 , 18)	7.48	(-24 , -18 , 10)	(-24 , -20 , 0)	10.2	(20 , -16 , 12)	(20, -16, 18)	6	(22 , -20 , 4)	(26, -20, -2)	7.21
Grog	(-20 , -10 , 20)	(-22 , -10 , 16)	4.47	(-22 , -28 , 6)	(-22 , -26 , 8)	2.83	(20 , -18 , 16)	(18, -18, 22)	6.32	(22 , -20 , -4)	(26, -16, 6)	11.49
Guru	(-18 , -22 , 22)	(-20 , -16 , 20)	6.63	(-26 , -18 , 4)	(-24 , -18 , 10)	6.32	(20 , -24 , 16)	(22, -18, 18)	6.63	(26 , -20 , 8)	(24, -16, -6)	14.7
Kefir	(-22 , -20 , 20)	(-24 , -16 , 14)	7.48	(-24 , -18 , 10)	(-24 , -18 , 10)	0	(22 , -12 , 16)	(22, -16, 14)	4.47	(22 , -10 , 4)	(24, -20, 8)	10.95
Kope	(-22 , -20 , 18)	(-22 , -18 , 20)	2.83	(-26 , -30 , 10)	(-26 , -24 , 6)	7.21	(20 , -22 , 22)	(20, -20, 22)	2	(24 , -20 , -4)	(24, -22, 4)	8.25
Manka	(-22 , -22 , 18)	(-20 , -20 , 20)	3.46	(-24 , -30 , 10)	(-24 , -28 , 6)	4.47	(24 , -20 , 20)	(26, -22, 12)	8.49	(26 , -16 , -4)	(26, -20, 8)	12.65
Maveri	(-24 , -18 , 14)	(-22 , -16 , 18)	4.9	(-24 , -20 , 10)	(-24 , -20 , 0)	10	(22 , -14 , 18)	(22, -14, 18)	0	(24 , -22 , 6)	(24, -20, 8)	2.83
Maya	(-22 , -18 , 16)	(-24 , -20 , 16)	2.83	(-22 , -22 , 12)	(-24 , -22 , 2)	10.2	(24 , -22 , 12)	(24, -24, 14)	2.83	(22 , -12 , -4)	(22, -12, -4)	0
Nia	(-24 , -18 , 20)	(-22 , -18 , 22)	2.83	(-26 , -20 , 0)	(-26 , -24 , 10)	10.77	(24 , -22 , 12)	(24, -18, 12)	4	(22 , -16 , -2)	(26, -22, 6)	10.77
Sander	(-22 , -20 , 16)	(-22 , -16 , 12)	5.66	(-26 , -20 , 2)	(-22 , -24 , 12)	11.49	(22 , -20 , 16)	(22, -24, 16)	4	(22 , -18 , 0)	(24, -16, -6)	6.63
mean			*5.15*			*7.41*			*4.7*			*8.89*

Supplementary Table S4. Individual peak coordinates in all 4 ROIs of each experiment per subject, and the Eucledian distance (mm) between them. Peak coordinates of the All acoustic stimuli vs. Silence contrast in all 4 ROIs (left and right primary and secondary cortices) in the two experiments (Speech sound and Voice identity processing). Distances (D) are calculated between each subject peak in one experiment and its corresponding peak in the other experiment.

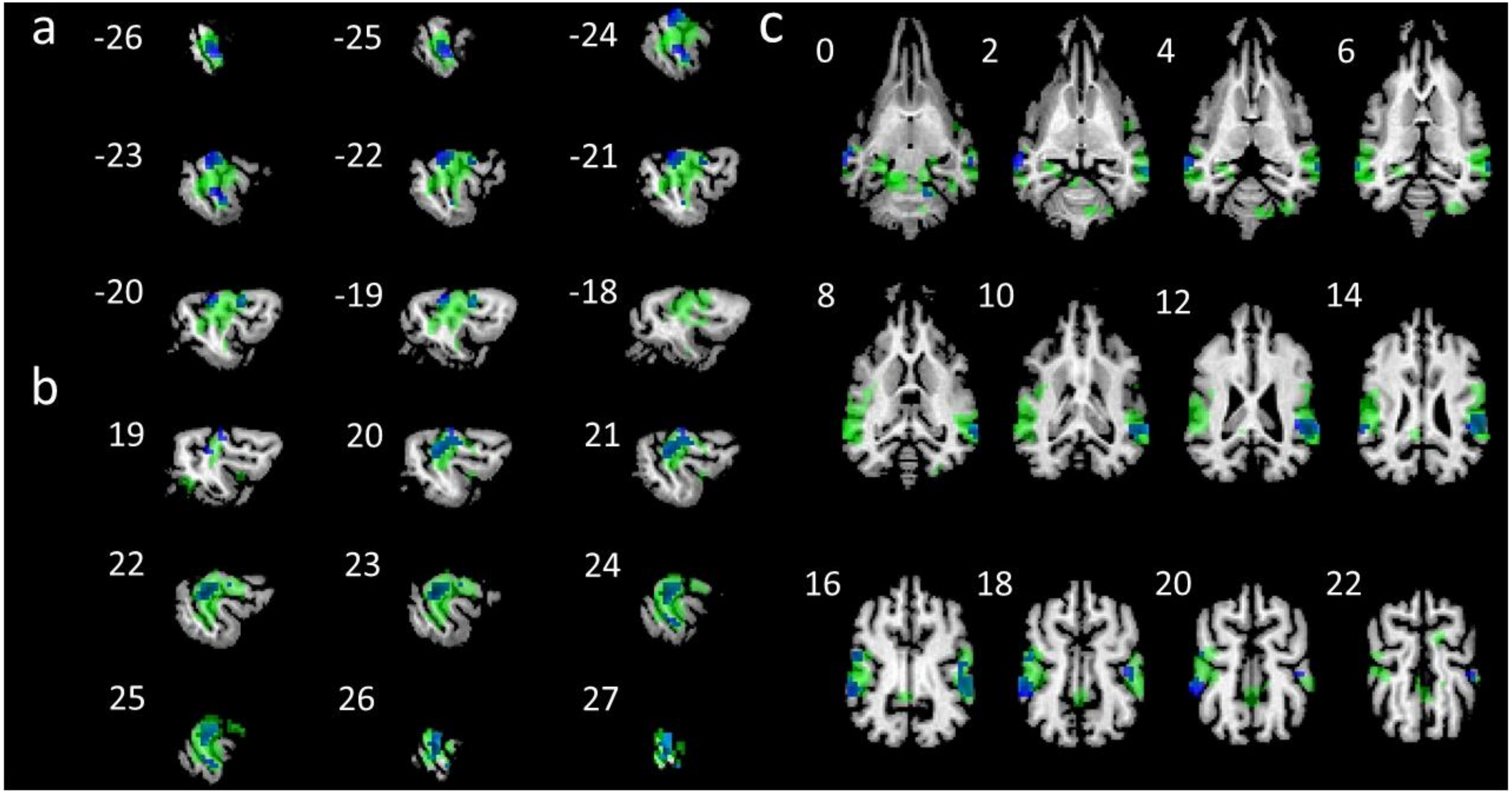

Supplementary Figure S1. Dog brain responses for the auditory stimuli in the speech sound processing (green) and the voice identity processing (blue) experiments. Group level activity maps for the All acoustic stimuli versus Silence contrast rendered on a template dog brain – shown here on sagittal slices in the left (a) and right (b) hemispheres as well as on (c) axial slices. Thresholds: $p < 0.001$, uncorrected for multiple comparisons at the voxel level.
